# Effect of the COVID-19 Outbreak on Pediatric Patients’ Admissions to the Emergency Department in an Italian Orthopedic Trauma Hub

**DOI:** 10.3390/children8080645

**Published:** 2021-07-27

**Authors:** Fabio Verdoni, Martina Ricci, Cristina Di Grigoli, Nicolò Rossi, Michele Davide Maria Lombardo, Domenico Curci, Riccardo Accetta, Marco Viganò, Giuseppe Maria Peretti, Laura Mangiavini

**Affiliations:** 1 IRCCS Istituto Ortopedico Galeazzi, 20161 Milan, Italy; verdonifabio@alice.it (F.V.); nicocurci73@gmail.com (D.C.); riccardo.accetta@grupposandonato.it (R.A.); marco.vigano@grupposandonato.it (M.V.); giuseppe.peretti@unimi.it (G.M.P.); 2 Residency Program in Orthopedics and Traumatology, University of Milan, 20122 Milan, Italy; martina.ricci@unimi.it (M.R.); cristina.digrigoli@unimi.it (C.D.G.); nicolo.rossi@unimi.it (N.R.); micheledavide.lombardo@unimi.it (M.D.M.L.); 3 Department of Biomedical Sciences for Health, University of Milan, 20133 Milan, Italy

**Keywords:** COVID-19, pediatric trauma HUB, outbreak, ER admissions, pediatric fractures, pediatric injuries

## Abstract

Background: The rapid diffusion of Coronavirus disease (COVID-19) in Northern Italy led the Italian government to dictate a national lockdown from 12 March 2020 to 5 May 2020. The aim of this observational cohort study is to analyze the differences in the number of pediatric patients’ admission to the Emergency Room (ER) and in the type and causes of injury. Methods: The pediatric population during the pandemic was compared to a similar group of patients admitted to the ER in 2019. Sex, age, triage color-code at admission, cause of trauma and presence of symptoms related to COVID-19 infection, discharge diagnosis and discharge modes were investigated. Results: The lockdown period led to a reduction of 87.0% in ER admissions with a particular decrease in patients older than 12 years old. Moreover, a trend towards more severe codes and an increase in home-related injuries were observed during the pandemic, whereas the diagnosis of fracture was less frequent in the pre-pandemic group (*p* < 0.0001). Conclusions: A significant decrease in the ER attendances was reported during the lockdown. A shift in the cause and type of injury was observed; only the most serious traumas sought medical care with a higher percentage of severe triage codes and fractures.

## 1. Introduction

Coronavirus disease (COVID-19) is a worldwide public health challenge, declared a pandemic by the World Health Organization on 12 March 2020 [1]. Northern Italy was the most affected area within the whole country and has been struggling with COVID-19 since the end of February 2020 [2]. The government dictated measures of national lockdown; in Italy, from 12 March 2020 to 5 May 2020, people could leave their homes only for proved necessity, no recreational activities or sport were allowed and schools were closed. Hospitals were overwhelmed with COVID-19 patients and most of the wards were converted into ICU or infectious diseases care units, while deferrable surgeries and outpatient visits were suspended. Two regional referral centers specialized in traumas and orthopedics emergencies were identified in Milan, Italy, as the hubs for minor traumas or non-deferrable elective orthopedic surgeries [3]. 

Indeed, during the COVID-19 crisis, the necessity to reduce the risk of virus exposure and transmission and the need to maintain the quality of care provided to critical patients forced the healthcare system to discourage unnecessary admissions to the Emergency Room (ER) related to minor traumas and other ailments. Therefore, a reduction in patient flow to the ER during the pandemic period was noticed, especially in the pediatric population. We performed an observational cohort study on the ER admission of pediatric population (<16) before and after lockdown. The aim of this study was to analyze the number of pediatric patients’ admission to the ER, the type and the possible causes of injury. 

## 2. Materials and Methods

We conducted a retrospective study of prospectively collected data. Data collection has been performed on two groups, according to the STROBE guidelines [4]. The Pandemic Group (Pandemic Group—PG) was composed of consecutive patients, aged 0–16 years, admitted to the ER of our Orthopaedic Trauma Hub Centre between 12 March and 5 May 2020, the lockdown period. The Non-Pandemic Group (NG) was composed of all the ER pediatric admissions between 12 March and 5 May 2019. The investigated variables were sex, age, triage color-code at admission, declared cause of trauma and presence of symptoms related to COVID-19 infection, discharge diagnosis and discharge modes. 

During ER admission, after checking for symptoms related to COVID-19 disease, the PG patients were assigned to a triage category by a nurse:

- White code: non-urgent patients;
- Green code: urgent but non-critical patients;
- Yellow code: fairly critical patients;
- Red code: very critical patients at danger of death.

Pediatric patients were defined as age ≤16 years old. 

After triage, patients were evaluated by the attending orthopedic surgeon that provided the appropriate treatment and the most adequate ER discharge mode.

The place where the trauma has occurred was also analyzed based on the clinical history:

- Injuries at home;
- Injuries at school;
- Injuries at play areas;
- Road injuries;
- Unknown: cause of injury was not reported.

Our Institutional Board did not require any ethical approval for this kind of study. All procedures performed in studies involving human participants were in accordance with the ethical standards of the institutional and/or national research committee and with the 1964 Helsinki declaration and its later amendments or comparable ethical standards.

### Statistical Analysis

The analyses were performed using Graphpad Prism v5.0 (GraphPad Software, San Diego, CA 92108, USA). Numerical data are presented as median and interquartile range; categorical variables are reported as absolute frequency and percentage. Distribution of continuous variables was assessed by Shapiro–Wilk test. In accordance with the results of this test, the difference between the groups were assessed by Student *t* test or Mann–Whitney test for normally and non-normally distributed variables, respectively; Fisher’s exact test was applied to categorical variables. Chi-square test for trends have been applied for ordinal categorical variables with more than 2 categories. A *p*-value < 0.05 was considered statistically significant. 

## 3. Results

Our main finding was the decrease in the number of pediatric patients admitted to ER during the pandemic period: the NG counts for 790 cases, whereas the PG includes 103 patients, showing a reduction of 87.0% in the admissions. Data are reported in Table 1; a statistically significant difference between the two groups was found in the mean age of patients, that decreased from 11.4 ± 3.4 (NG) to 8.6 ± 4.6 years (PG) (*p* < 0.0001); in particular, the decrease relates to the percentage of patients older than 12 years in 2020, from 41.8% in NG to 22.3% in PG (*p* < 0.0001; OR 0.40, CI 95%: 0.25–0.65) (Table 2). 

**Figure 1:**
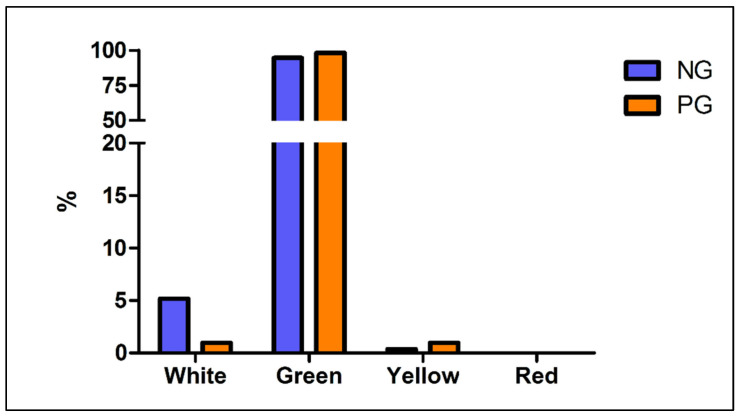
Triage code at ER admission: differences between NG and PG. Legend: NG: Non-pandemic Group PG: Pandemic Group.

**Figure 2:**
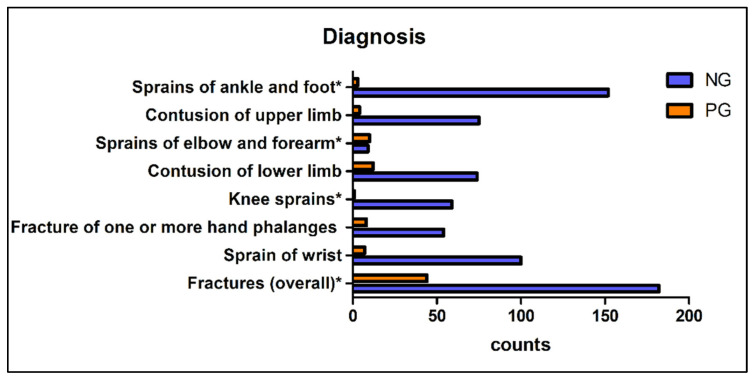
Most frequent diagnosis: differences between NG and PG. * = statistically significant difference. Legend: NG: Non-pandemic Group PG: Pandemic Group.

Regarding the triage code assigned at admittance, a trend toward more severe codes (green and yellow) in the PG compared to NG was recorded (*p* = 0.039) (Figure 1); the diagnosis of fracture was less frequent in NG (*p* < 0.0001) with an Odds Ratio of 2.78 (CI 95%: 1.75–4.09), while the percentage of contusions changed from 19.1% in NG to 13.6% in PG, though no significant difference was detected (*p* = 0.22; OR= 0.67 CI95%: 0.37–1.20). Additionally, a change in the most common diagnoses was noticed: in the NG prevailed ankle sprains (14.2%), forearm fractures (9.4%) and sprains of the interphalangeal joints (8.0%), whereas in PG, forearm fracture was the most frequent trauma (26.2%), followed by elbow contusions (7.2%) and fingers fractures (6.8%). Complete data are reported in Figure 2.

**Figure 3:**
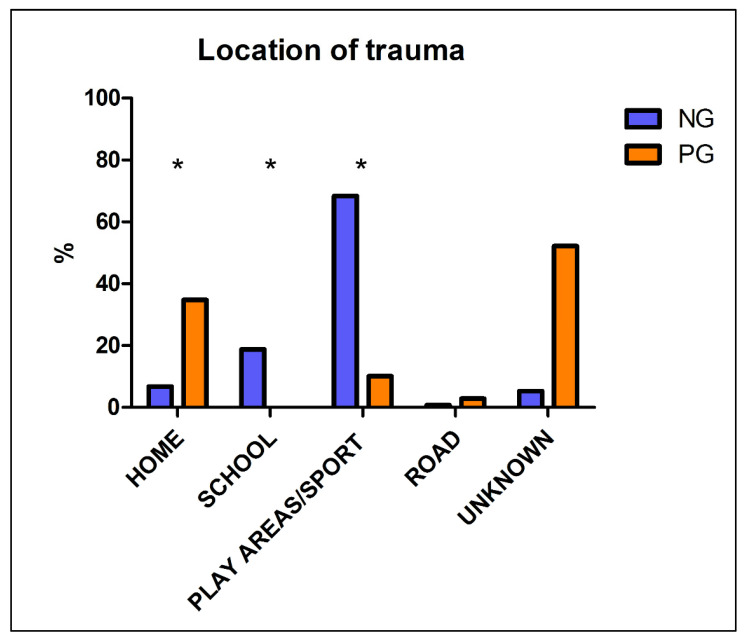
Place of traumas, differences between NG and PG. * = statistically significant difference, *p* < 0.0001. Legend: NG: Non-pandemic Group PG: Pandemic Group.

As far as the location of injuries, data were available for 474 patients out of 790 (60%) for the NG and for 69 patients out of 103 (67%) for the PG. As expected, during the pandemic period injuries at home were far more frequent (34.8% compared to 6.8% in NG), whereas in 2019, traumas mostly occurred during sport activities or at playgrounds (68.4%), followed by injuries at school (18.8%); all these differences are highly statistically significant (*p* < 0.0001). Detailed results are shown in Figure 3.

Surprisingly, no cases of suspected or confirmed COVID-19 infection were recorded in the PG at the investigated Emergency Room.

## 4. Discussion

Over the last few decades, ERs have known an important growth in patient flow. Thus, “Observatoire Regionale des Urgences Champagne—Ardennes” organization reported an increase in the number of admissions to the ERs in the Champagne-Ardennes state of France of 6.43% per year from 2008 to 2013 [5].

During the outbreak of COVID-19, patients were forced to postpone regular check-ups and non-urgent clinical or surgical procedures to avoid risks of COVID-19 transmission due to the overcrowding of the ERs and of the departments. At the same time, the government imposed a total lockdown in Italy and obligated people to stay at home except for proved necessities. Schools of any grade were closed and sport activities were prohibited. Our findings demonstrate that the lockdown led to 87% reduction in the overall ER pediatric patient flow of our Regional Trauma Hub. In normal conditions, unnecessary admissions contribute to the ER congestion with a long length of stay. Nevertheless, these issues resolved spontaneously during the pandemic. A similar decrease in ER patient’s flow was reported in Canada, Taiwan and Hong Kong during the SARS epidemic (2003–2004), and this may be attributed to people’s perception of the ER as a possible source of infection. As reported by Huang et al. [6], at the peak of the SARS epidemic, the reduction in daily ER visits reached 51.6% of pre-epidemic numbers (*p* < 0.01). In pediatric patients, the maximum mean decreases in number of visits were 80.0% (*p* < 0.01), 57.6% (*p* < 0.01) and 40.8% (*p* < 0.01), respectively. Moreover, this reduction persisted 3 months after the end of the epidemic. Man et al. [7] displayed a significant drop in the overall ER attendance following the outbreak of SARS; in particular, the trauma rate was significantly lower in 2003 than in 2002 (*p* = 0.03) due to the fear of virus exposure. In addition, during the SARS spread, as well as during the COVID-19 outbreak, most people preferred to avoid crowded areas; thus, recreational or sporting activities may have been less popular then before. Consequently, a change in community behavior may also explain the drop in ER admission reported in the present study. 

Moreover, Bhuvaneswari et al. [8] reported that the most common age group injured at home included patients younger than 12 years and toddlers. Similarly, our study demonstrates an important reduction of patients’ age during the lockdown. As a matter of fact, we found an important reduction of patients older than 12 years old who visited the Emergency Department during the COVID-19 outbreak (41.8% in 2019 vs. 22.3% in 2020) (Table 2). This finding may be related to several elements. First, younger children are less aware of the risks of injury and they probably have a similar behavior during outdoor or indoor activities. Thus, they are exposed to traumas even in a domestic context. Furthermore, the closure of schools and play areas constricted at home many older children. Therefore, older children radically changed their behaviors, becoming more focused on board games, computer games and videogames, as they were not allowed to meet with their friends and play outdoors. This clearly represents a difference in the risk of injuries since all these indoor recreational activities did not expose them to traumas. Lastly, older children are more keen on competitive sports activities and contact sports that were prohibited during the national lockdown, and this clearly can explain the reduction of ER admissions of these subset of pediatric patients. 

Farrell et al. [9] reported that during the SARS outbreak in 2003, ER visits declined by 21% (95% CI, 18–24%) over the 4-week study period. Conversely to what the present study shows, those authors found the greatest reduction involves both infant and toddler visits (69%; 95% CI, 58–79%) and these data did not recover the following year. This difference might be explained by the fact that our data are relative only to pediatric admissions in a Trauma Hub center specialized only in Orthopaedic surgery. Indeed, COVID-19 disease in neonates, infants and children has been reported to be significantly milder than their adult counterparts. Similarly, all the reported neonatal cases have been mild [10]. Concerning admissions to ER in our Center, no cases of COVID-19 were registered in children, whereas many adult patients diagnosed with COVID-19 were hospitalized at our institute. 

During the COVID-19 pandemic, a spike in the purchase of home play equipment and trampolines has been registered. Consequently, the lockdown per se did not prevent all injuries [9]. Regarding the place and causes of trauma, Prakash et al. [11] reported that up to 63.9% children attending ER in ordinary times sustained injuries at home, followed by road accidents (26.2%), whereas school and play areas accounted only for 8.8% of traumas. Conversely, Sephton et al. reported a change in mechanism of injuries during the pandemic period. Indeed, authors showed that the proportion of sports-related injuries during the lockdown period fell from 6.2% to 3.6% [12]. Similarly, the present study demonstrated a big shift from non-domestic traumas (including both scholastic, sport and play areas injuries) to injuries occurred at home in the NG in comparison with the PG (respectively 6.8% and 34.8%). More specifically, our study showed 0% of scholastic traumas during the period of lockdown and only 10% of play area injuries, whereas in 2019 they counted for 18.8% and 68.4% of traumas, respectively. This shift is obviously due to the banning of both outdoor activities and sports performed in gyms and swimming pools. These measures led to a drop of patients presenting for non-urgent chronic reasons, sports-related injuries (sprains, contusions, dislocations) and minor road accidents. Therefore, fewer minor traumas such as sprains of knee reached our ER, as expected, and this finding explains the decreased percentages of non-urgent codes and a statistically significant tendency towards more serious triage codes in the PG. Moreover, we found that the fracture diagnosis was more frequent in the PG in comparison with the NG, confirming that only the most severe injured patients sought medical attention during the pandemic period. In addition, forearm fractures and elbow contusions were the most frequent traumas in the PG, consistently with the younger age of ER admissions as these type of injuries are more frequent in this subset of pediatric patients [13]. 

A recent study also reported an increase in domestic violence and child abuse, particularly in dysfunctional families during the pandemic period. This event might be related to restrictions, isolation, higher level of anxiety and stress with a relevant impact on risks of developmental delay and behavioral problems [14]. Nevertheless, our study did not show any increase in domestic violence and child abuse rate, probably because our institution is mainly an Orthopedic Trauma Hub and it lacks a psychological or psychiatric support for children and adolescents; thus, suspected child abuses were not recorded. A more extensive analysis involving other hospitals with a pediatric psychiatric service may confirm the results shown by Boo et al. [14]. 

The conclusions drawn from this study rely on data about an Orthopedic Trauma Hub that includes also a Pediatric Orthopedics service and may be different from the flow in other hospitals. Thus, we cannot comment on the pediatric patients’ flow in the ER due to ailments other than traumas. Nevertheless, this is the first study revealing the epidemiologic effects that COVID-19 pandemic and lockdown measures had on pediatric patients’ flow in an emergency department. 

Moreover, our study indirectly demonstrated that the vast majority of ER admissions in normal conditions is due to non-urgent or deferrable conditions. Thus, this evidence-based analysis is fundamental to improving the strategies of care of the National Health System, to better employ the available resources and to reduce overcrowding in the ER that usually leads to long waiting time for patients and the risk of a lower standard of care. 

## 5. Conclusions

A significant drop in the overall ER attendances in a Trauma Hub center was reported after the outbreak of COVID-19 pandemic. The fear of virus exposure in hospital undoubtedly acted as a significant deterrent. However, it is likely that the community precautions adopted during the lockdown, namely school closures and decreased sport activities, resulted in fewer injuries; thus, only the most serious traumas sought medical care, resulting in a higher percentage of severe triage codes and fractures.

## Figures and Tables

**Table 1:** Demographic differences between NG and PG.

	NG (2019)	PG (2020)
Number of patients *	790	103
Sex (Males/Females)	454/336 (ratio 1.35)	55/48 (ratio 1.15)
Mean age (years) *	11.4 ± 3.4	8.6 ± 4.6

* = statistically significant difference, *p* < 0.0001.

**Table 2:** Patients divided by age groups admitted to ER: differences between NG and PG.

Age (Years)	0–2	2–6	6–12	>12	Mean Age	SD	Median Age
NG (2019)	2.3%	6.5%	49.5%	41.8%	11.4	3.4	12
PG (2020)	14.6%	15.5%	47.6%	22.3%	8.6	4.6	9

## Data Availability

Data supporting reported results can be found at this link: https://osf.io/k2wh5/?view_only=e59648fbba854840b32ca3988736da6a (accessed on 26 July 2021).
